# Recent Progress on High-Efficiency Perovskite/Organic Tandem Solar Cells

**DOI:** 10.3390/nano15100745

**Published:** 2025-05-15

**Authors:** Kelei Wang, Jiana Zheng, Runnan Yu, Zhan’ao Tan

**Affiliations:** Beijing Advanced Innovation Center for Soft Matter Science and Engineering, Beijing University of Chemical Technology, Beijing 100029, China; w1370348740@163.com (K.W.); 15258105037@163.com (J.Z.); tanzhanao@mail.buct.edu.cn (Z.T.)

**Keywords:** perovskite solar cells, organic photovoltaic cells, tandem device, device engineering

## Abstract

Perovskite/organic tandem solar cells, as a next-generation high-efficiency photovoltaic technology, integrate the tunable bandgap characteristics of perovskite materials with the broad spectral absorption advantages of organic semiconductors, demonstrating remarkable potential to surpass the theoretical efficiency limits of single-junction cells, enhance device stability, and expand application scenarios. This architecture supports low-temperature solution processing and offers tunable bandgaps, lightweight flexibility, and ecofriendly advantages. This review systematically summarizes research progress in this field, with a primary focus on analyzing the working principles, performance optimization strategies, and key challenges of the technology. Firstly, the article discusses strategies such as defect passivation, crystallization control, and suppression of phase separation in wide-bandgap perovskite sub-cells, offering insights into mitigating open-circuit voltage losses. Secondly, for the narrow-bandgap organic sub-cells, this paper highlights the optimization strategies for both the active layer and interfacial layers, aiming to improve spectral utilization and enhance power conversion efficiency. Additionally, this paper emphasizes the optimization of optical transparency, electrical conductivity, and energy level alignment in the recombination layer, providing theoretical guidance for efficient current matching and carrier transport.

## 1. Introduction

Over the past decade, single-junction perovskite solar cells (PSCs) have achieved power conversion efficiencies (PCEs) as high as 27%, while organic photovoltaics (OPV) have reached 21.0% [1]. However, the efficiency of single-junction solar cells is fundamentally constrained by the Shockley–Queisser limit (~33.7%) [2,3], due to several intrinsic loss mechanisms: (1) limited spectral absorption: photons with energies below the bandgap cannot be harvested; (2) thermalization loss: photons with energies above the bandgap dissipate excess energy as heat during carrier relaxation; and (3) insufficient charge transport: despite a high absorption coefficient, short carrier lifetimes and low mobilities necessitate ultra-thin films, limiting complete photon harvesting even at peak absorption. To overcome these limitations and further improve device performance, tandem solar cell (TSC) architectures have emerged as a promising strategy to enable broader spectral utilization and suppress energy losses associated with hot carrier relaxation [4]. According to theoretical calculations by Shockley et al., the efficiency limit of TSCs can exceed that of single-junction counterparts, potentially reaching 42%.

Perovskite materials possess a general ABX_3_ crystal structure, where the A-site is occupied by inorganic or organic cations such as Cs^+^, CH_3_NH_3_^+^, or HC(NH_2_)_2_^+^. The size and stability of the A-site cations are crucial in determining the crystallinity and long-term phase stability of the perovskite lattice. The B-site consists of metal cations, such as Pb^2+^ and Sn^2+^, while the X-site is composed of halide anions (Cl^−^, Br^−^, I^−^). The coordination between B-site cations and X-site anions forms a stable BX_6_ octahedral structure, which serves as the structural backbone of the perovskite lattice [5]. By modulating the elemental composition at the A, B, and X sites, the optical bandgap of perovskite materials can be finely tuned in the range of 1.2–2.3 eV, enabling compatibility with various tandem device architectures [6,7,8]. Consequently, perovskite-based TSCs exhibit significant development potential due to their compositional versatility and tunable optoelectronic properties [9]. Currently, perovskite TSCs are typically classified into perovskite/silicon-based tandem cells, all-perovskite tandem cells, and perovskite/organic tandem solar cells (PO-TSCs). Among these, all-perovskite TSCs have recently demonstrated a PCE of 30.1%, gradually approaching the previously reported efficiency of 34.6% for perovskite/silicon-based solar cells. However, perovskite materials remain highly susceptible to environmental factors such as moisture, oxygen, and prolonged light exposure, which can lead to decomposition when directly exposed to these environments, thereby reducing the stability of PSCs. Narrow-bandgap OPV, when employed as sub-cells in TSCs, possesses unique advantages and application potential. The bandgap of narrow-bandgap OPV typically ranges from 1.1 eV to 1.4 eV, enabling them to absorb photons in the near-infrared region (NIR). When integrated with wide-bandgap perovskite solar cell (PSC) sub-cells, they facilitate more comprehensive solar spectrum utilization and enhance overall photon harvesting efficiency. Moreover, the intrinsic tunability of energy levels and absorption profiles in organic materials achievable via molecular design and synthetic chemistry enables precise bandgap engineering and energy level alignment with PSC sub-cells, leading to enhanced PCE. Additionally, the ability of OPV to perform solution processing represents a significant advantage when integrated with PSC sub-cells. Fabrication methods such as spin coating and spray coating can be employed, leveraging this characteristic to unify the manufacturing process and reduce production costs.

This review focuses on the rapid advancements in PO-TSCs in recent years. Drawing on the latest research progress, it systematically introduces effective strategies and technologies for enhancing the performance of tandem devices from three critical dimensions: wide-bandgap perovskite sub-cells, interconnecting/recombination layers, and narrow-bandgap organic sub-cells. Additionally, it provides prospective insights into the persistent challenges and technical bottlenecks confronting this field, aiming to offer a comprehensive reference for advancing the future development and industrialization of these hybrid tandem devices.

## 2. Architecture and Design of TSCs

**Figure 1:**
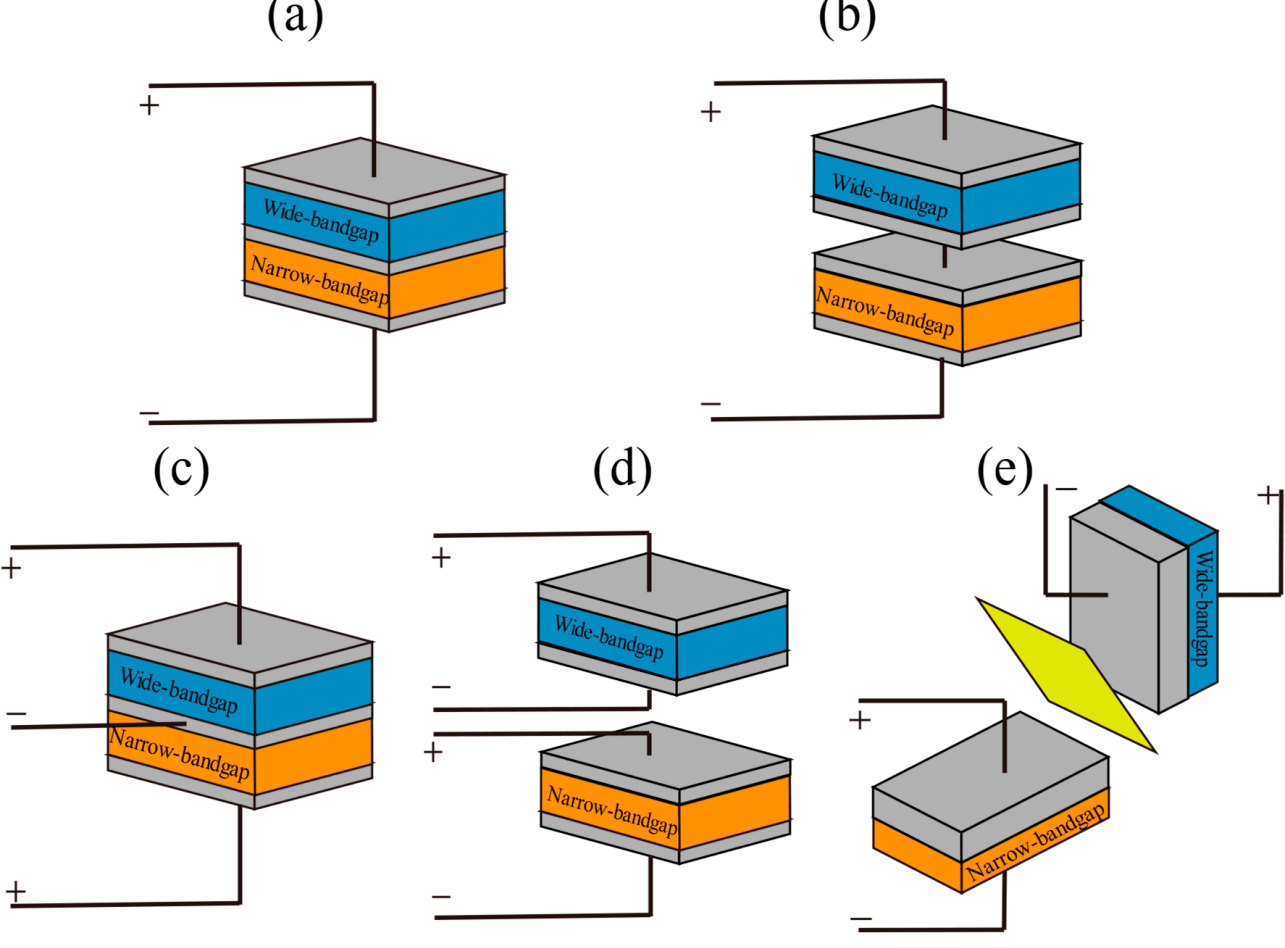
Tandem device structures: (**a**) 2T monolithic integrated structure; (**b**) 2T mechanically stacked structure; (**c**) 3T monolithic integrated structure; (**d**) 4T monolithic integrated structure; and (**e**) 4T mechanically stacked structure.

TSCs typically consist of a wide-bandgap front sub-cell (B) and a narrow-bandgap back sub-cell (T), connected by an intermediate layer to form either a series or parallel structure. This enables the segmented utilization of the solar spectrum, thereby ensuring device performance is not limited by the thickness of a single active layer and further enhancing the optoelectronic performance of the device [10,11]. Currently, tandem device structures mainly include two-terminal (2T), three-terminal (3T), and four-terminal (4T) configurations. Combining stacking methods and connection techniques, tandem structures can be further classified into the following five types, as shown in Figure 1.

**Figure 2:**
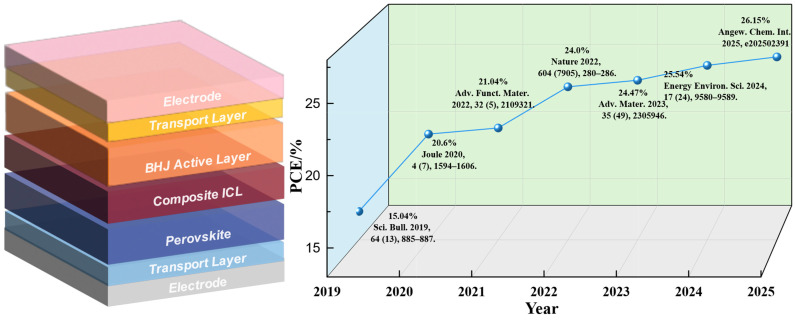
2T structure and the recent development of PO-TSCs [13,14,15,16,17,18,19].

The 2T structure is typically categorized as a monolithic integrated structure. In a 2T monolithic integrated structure, the top and bottom sub-cells are interconnected via ICL, forming a single monolithic device. This structure requires current matching between the two sub-cells, which necessitates identical current generation from both sub-cells to ensure high device efficiency. This requirement necessitates precise tuning of the absorption ranges of the sub-cells during design to ensure efficient utilization of light energy across different spectral regions. However, due to current-matching constraints, the overall performance may be limited by the sub-cell with the lower current output. From a commercialization perspective, the 2T monolithic structure simplifies the fabrication process by depositing both sub-cells on the same substrate, thereby reducing production costs. Additionally, the 2T monolithic structure requires only two output terminals, which facilitates large-scale integration and manufacturing. Implementing series and parallel connections and scribing cutting techniques for single-junction photovoltaic cells can effectively reduce series resistance losses in large-area modules, thereby further enhancing system efficiency. Compared to the 2T monolithic structure, the 2T mechanically stacked structure offers greater flexibility. It consists of two independently fabricated sub-cells connected in series via wires, eliminating the need for interfacial interconnecting layers and allowing individual optimization of each sub-cell. This structure allows for greater manufacturing flexibility, enabling the selection and optimization of different materials tailored to specific requirements. However, the 2T mechanically stacked structure faces several challenges. To achieve high device efficiency, this structure typically requires the use of multiple transparent conductive layers. The presence of these layers inevitably induces optical losses, and their fabrication processes are relatively complex, thereby increasing production costs [12]. Figure 2 illustrates a typical 2T PO-TSC structure. In this device architecture, the current flows in series within the same circuit, which substantially enhances the overall energy conversion efficiency. Furthermore, the bandgap alignment of each sub-cell is optimized to absorb solar radiation more effectively across a wide range of wavelengths, thereby reducing optical losses.

The development of 2T PO-TSCs spans more than a decade. In 2015, Chen reported a 2T PO-TSC [20]. They optimized the initial crystallization process and enhanced the uniformity of the perovskite film by incorporating the small-molecule additive BmPyPhB into the wide-bandgap perovskite CH_3_NH_3_PbI_3_. Furthermore, crystal growth was accelerated via a solvent-washing technique, ultimately achieving a PCE of 9.1% in single-junction PSCs. The 2T PO-TSCs fabricated in combination with the polymer PBSeDTEG8 achieved a power conversion efficiency of 10.23%. In 2019, Zeng employed the all-inorganic perovskite CsPbI_2_Br as the front sub-cell of 2T PO-TSCs to capture high-energy photons, thereby effectively extending the device’s spectral absorption range. By utilizing PTB7-Th:COi8DFIC as the absorber layer for the rear sub-cell, the resulting 2T PO-TSCs achieved a PCE of 15.04% and a *V*_OC_ of 1.71 V under reverse scanning conditions [13]. In 2022, Chen et al. reported that the passivation of nickel oxide hole-transporting layers with benzylphosphonic acid leads to the suppression of interfacial recombination, resulting in 2T PO-TSCs that achieved a PCE of 23.6% [21]. In 2024, K.O. Brinkmann et al. fabricated an ultra-thin (1.5 nm) indium oxide intermediate layer using atomic layer deposition [22]. By integrating high-efficiency PSC sub-cells with OPV sub-cells, they successfully manufactured 2T PO-TSCs boasting a PCE of up to 24.0%. Zhang proposed a pseudo-ternary halide alloy perovskite model based on I/Br/SCN and conducted a systematic investigation of crystallization dynamics, examining the macroscopic crystallization morphology, microscopic lattice composition, and photovoltaic performance of wide-bandgap perovskites. This comprehensive approach led to the fabrication of highly efficient and photostable 2T PO-TSCs with a world record-certified power conversion efficiency of 25.06% [23].

In contrast to conventional multilayer photovoltaic designs, 2T TSCs employ a shared transparent intermediate electrode; this breakthrough design not only significantly reduces energy losses associated with interface reflection and photon absorption but also eliminates redundant electrical interconnection layers inherent in conventional multilayer architectures, thereby effectively mitigating additional electrical losses due to contact resistance and parasitic absorption. This integrated electrode design enables the 2T tandem structure to maximize the utilization of the solar spectrum: wide-bandgap PSC sub-cells preferentially absorb high-energy visible light, while narrow-bandgap OPV sub-cells efficiently capture near-infrared light, thereby achieving seamless spectral integration through synergistic operation. Moreover, the 2T structure—via its shared-electrode current matching mechanism—ensures synchronized photocurrent output from the two sub-cells in series, thereby circumventing the efficiency bottleneck caused by current mismatch in conventional multilayer cells. This compact device design not only reduces manufacturing complexity but also significantly lowers production costs by minimizing material usage and processing steps. For example, an ultra-thin transparent oxide intermediate layer fabricated via atomic layer deposition serves both as an efficient charge recombination layer and as a barrier against solvent-induced degradation of the underlying layers, thereby achieving dual optimization of optical and electrical performance. In addition, the process compatibility of the 2T structure provides technical assurance for large-scale production. Its solution processing properties permit the use of low-cost techniques such as spin coating, spray coating, or printing, while the self-aligning design of the shared electrode obviates the need for the complex photolithographic alignment steps typically required in conventional multilayer cells. This design, which combines high efficiency with manufacturability, is emerging as a critical pathway to surpass the Shockley–Queisser efficiency limit for single-junction cells, thereby ushering in a new technological paradigm for the commercialization of PO-TSCs. With ongoing advancements in material stability and interface engineering, the 2T structure is garnering increasing attention [22,23,24,25,26,27].

The 3T structure is analogous to the 2T structure in that both consist of monolithically integrated front and back sub-cells. However, their interconnection strategies differ. In the 3T structure, the sub-cells are interconnected via a transparent electrode instead of a tunneling layer, thereby circumventing the current-matching challenges inherent in 2T. The output terminals of the two sub-cells are merged into a single common output terminal. Consequently, the overall current density in the 3T structure equals the sum of the individual current densities from the front and back sub-cells, while the voltage is constrained by the sub-cell with the lower voltage. This design not only provides enhanced flexibility by eliminating current-matching limitations but also improves overall energy conversion efficiency [28].

**Figure 3:**
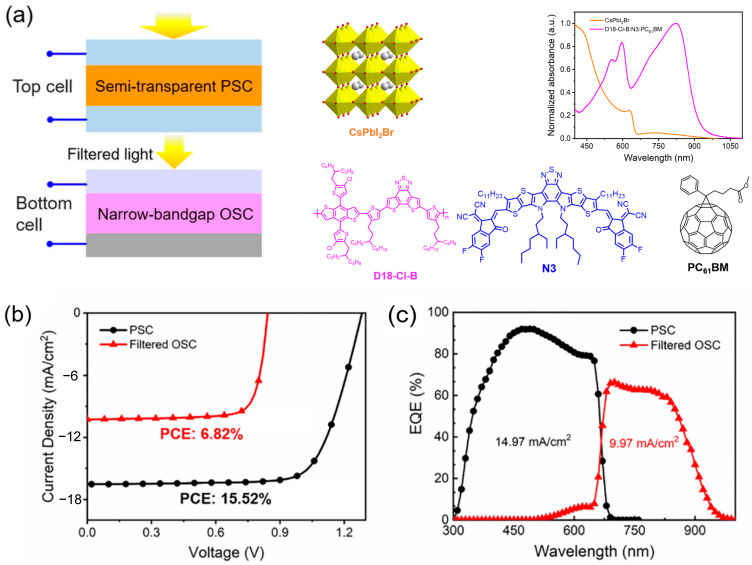
(**a**) Schematic of the PO-TSCs and the active layer materials used in the sub-cells; (**b**) *J-V* curves of the PSC and OPV sub-cells; and (**c**) EQE spectra of the PSC and OPV sub-cells. Strategies for enhancing PO-TSC performance. Reprinted with permission from [33], Copyright 2022, Springer Nature.

Research on 4T PO-TSCs remains less extensive than that on 2T structures, which are typically classified into mechanically stacked and spectral-splitting structures. The 4T mechanically structured design is analogous to the 2T mechanically stacked design but differs in that the sub-cells’ output terminals are not connected in series. Instead, they operate independently, yielding four distinct output terminals. This structure similarly avoids reliance on interconnecting layers and permits separate fabrication of the sub-cells. However, it necessitates an additional transparent conductive layer. Moreover, the 4T structure enables independent optimization of current and voltage across a broader operational range, thereby relaxing the current-matching constraints. These attributes afford the structure distinct advantages in both scientific investigation and performance optimization, as well as significant potential for commercialization [29,30,31,32]. The 4T spectral-splitting structure is derived from the 4T mechanically stacked structure. As shown in Figure 3, this structure offers enhanced device-level flexibility since each sub-cell can be optimized independently, free from mutual interference, and without requiring an additional transparent conductive layer. Nevertheless, reliance on costly dichroic mirrors curtails its commercialization potential.

The advantages of the 4T structure are primarily evident in its capacity for performance optimization. In this design, the front and back sub-cells are independently connected, enabling each to be optimized separately without the limitations imposed by current matching. Consequently, the 4T structure provides a broader scope for optimizing current, voltage, and efficiency. This structure facilitates efficient energy conversion across diverse spectral conditions, exhibiting improved stability and performance, especially under nonideal illumination. Liu et al. selected wide-bandgap all-inorganic semi-transparent PSCs as the front sub-cell and OPV as the back sub-cell to fabricate a 4T PO-TSC with a PCE of 22.34% [33]. Figure 3 illustrates the structural schematic of the 4T PO-TSC. The perovskite material used was all-inorganic CsPbI_2_Br, which has a narrow absorption range, primarily absorbing solar photons below 600 nm. The organic active layer consists of donor material, D18-Cl-B, and two acceptor materials, N3 and PC_61_BM. As demonstrated by the UV-Vis absorption spectra, the narrow-bandgap organic photovoltaic materials exhibit complementary absorption characteristics when compared to the perovskite material. This complementary absorption is further validated by the EQE spectra of the two sub-cells. Therefore, in this 4T tandem solar cell, sunlight filtered through the semi-transparent PSCs can be effectively utilized by the OPV. However, the 4T structure is not without its challenges. One of the main issues lies in the requirement for an additional transparent conductive layer, which increases both cost and technical complexity. The transparent conductive layer serves to connect the front and back sub-cells, ensuring current conduction. Nevertheless, its fabrication process is complex and requires high-precision equipment such as vacuum sputtering or atomic layer deposition (ALD) techniques. These high-cost devices limit the large-scale production and commercialization of 4T structures.

### 2.1. Optimization of Wide-Bandgap PSCs

Wide-bandgap PSCs are typically constructed with an inverted p-i-n device architecture and serve as the front sub-cells in TSCs, with their performance directly impacting the overall efficiency and stability of the TSC devices [34,35]. According to device modeling, the bandgap of PSCs suitable for TSCs ranges from 1.7 to 1.9 eV [36]. To address issues encountered in the application of wide-bandgap PSCs for TSCs such as defect-induced non-radiative recombination, low carrier transport efficiency, and electrical losses (particularly in *V*_OC_) caused by current mismatch, various strategies have been developed, including additive engineering, compositional engineering, and surface and interface passivation, to tune the properties of wide-bandgap perovskites and thereby enhance PCE [37,38,39,40,41,42,43].

**Figure 4:**
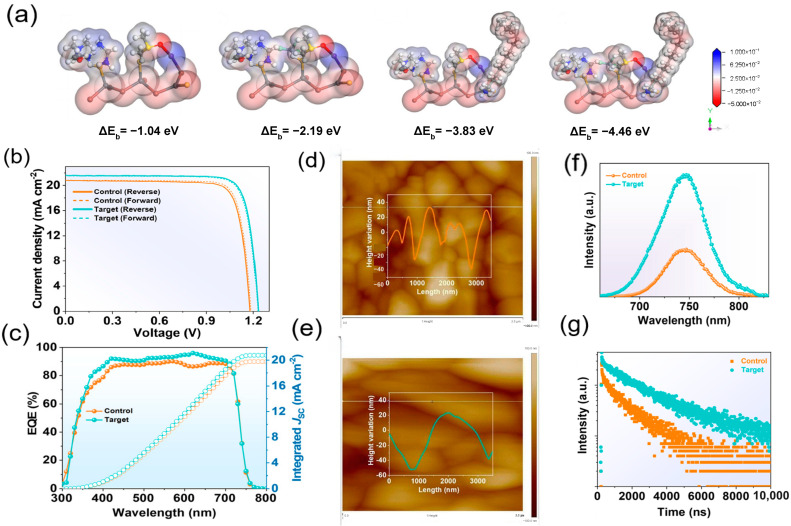
Bulk trap passivation in wide-bandgap perovskite films. (**a**) MEP distribution of the perovskite-OAmI-CHCl3 complex; (**b**,**c**) device *J-V* and EQE curves; (**d**,**e**) AFM topography images; and (**f**,**g**) steady state PL and TRPL spectra. Reprinted with permission from [44], Copyright 2024, Royal Society of Chemistry.

**Figure 5:**
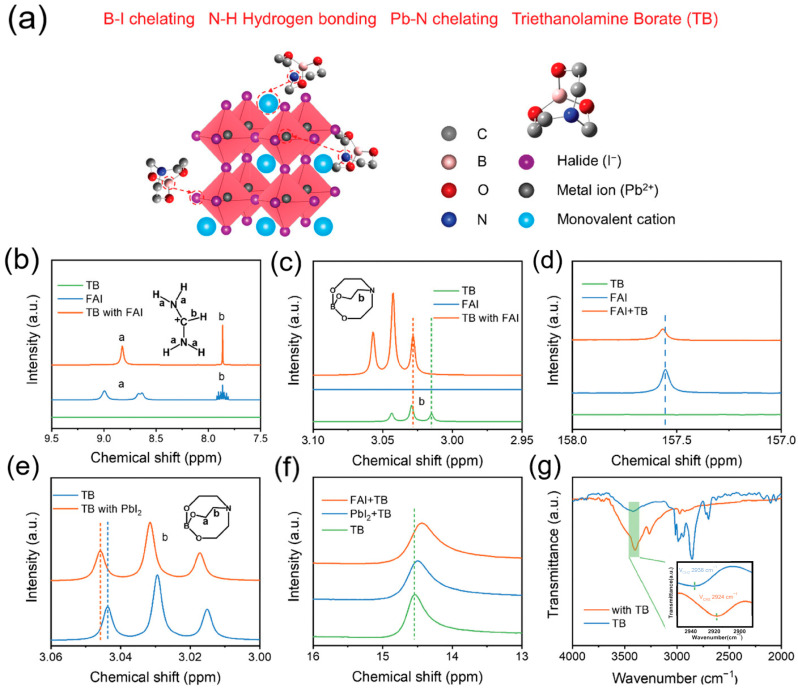
Chemical interaction characterization. (**a**) Schematic illustration of the proposed interaction mechanism between TB and perovskite. (**b**,**c**) 1H-NMR spectra of TB, FAI, and TB containing FAI solutions (DMSO-d6). In the figure, a and b are the resonance splitting peaks of hydrogen. (**d**) 13C-HMR spectra of TB, FAI, and TB containing FAI solutions (DMSO-d6). (**e**) 1H-NMR spectra of TB and TB containing PbI_2_ solutions (DMSO-d6). (**f**) 11B-NMR spectra of TB, TB containing FAI, and TB containing PbI_2_ solutions (DMSO-d6). (**g**) FTIR spectra of pure TB and TB with perovskite films. Reprinted with permission from [52], Copyright 2024, Wiley.

**Figure 6:**
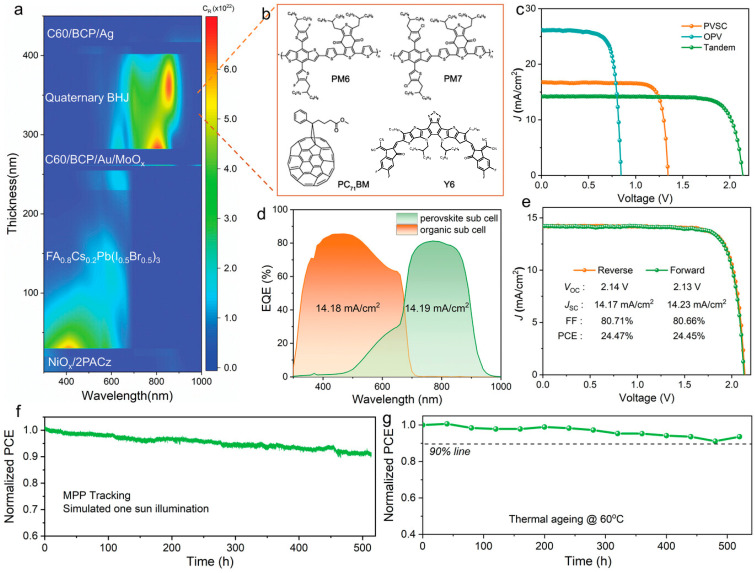
(**a**) Simulated charge generation rate (CR) distribution within the PO-TSC as a function of wavelength; (**b**) molecular structures of donors and acceptors of quaternary BHJ; (**c**) *J-V* curves of champion perovskite sub-cells, organic sub-cells, and tandem solar cells at reverse scan; (**d**) EQE spectra of two sub-cells in PO-TSC; (**e**) *J-V* curves of champion PO-TSCs at forward and reverse scan; (**f**) maximum power tracking of PO-TSCs under simulated one sun illumination; and (**g**) thermal stability of POTSCs under 60 °C in N_2_ atmosphere. Reprinted with permission from [17], Copyright 2023, Wiley.

(1) Defect passivation and carrier lifetime enhancement: In wide-bandgap PSCs (e.g., FA_0.8_Cs_0.2_Pb(I_0.6_Br_0.4_)_3_), the incorporation of Br exacerbates lattice distortion, resulting in a proliferation of dangling bonds and vacancy defects. Additives passivate these defect sites via chemical bonding or physical filling, reducing non-radiative recombination rates by one to two orders of magnitude and thereby increasing the device *V*_OC._ Ma et al. proposed a one-step dual-additive passivation strategy [44], in which oleylammonium iodide (OAmI) and chloroform (CHCl_3_) are introduced into the perovskite precursor solution to effectively reduce bulk defects and significantly suppress non-radiative recombination; Figure 4 presents data on bulk trap passivation in the perovskite films. The optimized perovskite films exhibit enlarged grain sizes and fewer grain boundaries, and the formation of 2D/3D heterostructures further enhances carrier-separation efficiency. In an inverted single-junction device based on NiO_x_, a PCE of 21.97% (certified 20.77%) and a *V*_OC_ of 1.25 V were achieved under one-sun illumination. Under indoor U30 illumination, the PO-TSCs attained a certified efficiency of 44.72% (*V*_OC_ = 1.069 V, FF = 82.3%), approaching the theoretical limit of 79.3%, and demonstrated long-term stability exceeding 800 h. Additionally, multifunctional fluorinated additives can be introduced to improve halide distribution uniformity and defect passivation. For example, Li et al. developed an 8-pentafluorobenzoxyquinoline (8-PFBQ) additive [45], in which the quinoline moiety anchors Pb^2+^ defects via strong coordination, while the pentafluorobenzyl group modulates organic halide distribution through anion–π and hydrogen bond interactions, synergistically enhancing crystal quality and compositional uniformity. This strategy markedly reduces non-radiative recombination losses, increasing the PCE of 1.67 eV wide-bandgap perovskite solar cells from 19.99% to 22.22%, while *V*_OC_ rises from 1.133 V to 1.243 V, and device stability doubles. This design offers a novel approach for defect management in wide-bandgap PSCs and the development of TSCs. Chen et al. introduced a bifunctional polymerizable additive, N-(3-(dimethylamino)propyl)-methacrylamide (DPM), significantly mitigating defect and ion migration issues in 1.86 eV wide-bandgap perovskite films [46]. During thermal annealing, DPM polymerizes in situ to form PDPM, whose dynamic hydrogen bond network effectively passivates grain-boundary defects and suppresses iodide migration, significantly reducing non-radiative recombination. Additionally, DPM stabilizes mixed cations (MA^+^/FA^+^) via hydrogen bonding, inhibiting condensation reactions in the precursor solution and substantially enhancing stability. The resulting single-junction wide-bandgap PSCs achieved a PCE of 18.19% and retained 84% of their initial efficiency after 1000 h of maximum power-point tracking. When integrated into PO-TSCs, the PCE further increased to 25.06%, demonstrating excellent long-term stability (T80 > 360 h). This study provides new strategies for defect control and stability enhancement in wide-bandgap perovskites.

(2) Phase separation suppression: Wide-bandgap perovskites, which contain higher bromide content than their narrow-bandgap counterparts, often exhibit halide segregation during operation. This phenomenon—driven by photoinduced iodide oxidation—leads to significant *V*_OC_ losses [47,48,49]. Wu et al. developed an anthraquinone-based redox mediator that selectively reduces iodine (I_2_) and oxidizes metallic lead (Pb^0^), effectively inhibiting phase separation in mixed-halide perovskites and significantly enhancing the stability and performance of wide-bandgap perovskite solar cells [50]. Furthermore, the mediator passivates defects via cation exchange (e.g., NH_4_^+^ and PEA^+^), enabling single-junction wide-bandgap PSCs to achieve a PCE of 19.58% and a high *V*_OC_ of 1.35 V, while retaining 95% of their initial efficiency after 500 h of maximum power-point tracking. On this basis, monolithic PO-TSCs achieved a PCE of 25.22% (certified 24.27%) and maintained 92% of their initial efficiency after 500 h of continuous operation. Dong et al. optimized the perovskite crystallization process by introducing dimethylammonium ions (DMA^+^), significantly suppressing phase separation and enhancing device stability 19. Studies show that the larger ionic radius of DMA^+^ (~2.17 Å) at the lattice A-site induces tilting of PbX_6_^4−^ octahedra, shortening the Pb-I bond length from 3.183 Å to 3.174 Å. The high dipole moment of DMA^+^ (4.2 D) further strengthens DMA–I interactions, transforming lattice strain from tensile (0.401%) to compressive (0.185%) and reducing lattice defects, as evidenced by a decrease in Urbach energy from 54.29 meV to 49.70 meV. DMA^+^ delays crystallization by forming a DMAPbI_1.8_Br_1.2_ intermediate phase, extending annealing time, and enabling a more complete lattice reaction. Optimized Cs_0.3_DMA_0.1_I_1.8_ films under 3000 mW cm^−2^ illumination and 1.8 V bias exhibited an increase in ion migration activation energy (E_a_) from 0.183 eV to 0.481 eV, significantly suppressing halide loss. Ultimately, PO-TSCs (0.062 cm^2^) achieved a certified efficiency of 26.15% (*V*_OC_ = 2.148 V) and retained 90% of their initial efficiency after 1350 h of maximum power-point tracking, validating the effectiveness of the lattice passivation strategy. Chen et al. proposed a cation-alloying strategy by introducing imidazolium (IA) to modulate the crystallization kinetics of mixed-halide perovskites, thereby addressing halide segregation and suppressing phase separation [18]. IA balances I^−^/Br^−^ nucleation rates through electrostatic and coordination interactions, homogenizing halide distribution and reducing electron defect density to 9.67 × 10^15^ cm^−3^ and hole defect density to 6.22 × 10^15^ cm^−3^. The optimized 1.79 eV wide-bandgap PSCs achieved a PCE of 19.50%, a *V*_OC_ of 1.35 V, and an energy loss of only 0.44 eV, approaching 90% of their Shockley–Queisser limit. DFT calculations indicate that strong IA-Pb^2+^ coordination (binding energy lowered by 0.33 eV) and surface defect passivation (iodine interstitial defect levels moved into the valence band) are key factors in performance enhancement. Upon integration with OPV sub-cells, the monolithic PO-TSCs achieved a PCE of 25.54%, illustrating a universal strategy for phase separation suppression via crystallization kinetics control and defect passivation.

(3) Crystallization Quality Optimization: An et al. proposed a crystallization kinetics modulation method using the multifunctional additive phenylethylammonium acetate (PEAAc), which markedly improves phase-distribution uniformity and defect management in mixed-halide perovskites [51]. The -NH_3_^+^ and -COO- groups in PEAAc interact with perovskite components via hydrogen bonds and coordination bonds: the ammonium anchors halide vacancies, while the carboxylate passivates undercoordinated Pb^2+^ and organic cation (e.g., FA^+^) defects, thereby synchronously suppressing both electron and hole traps. This synergistic action delays rapid Br^+^ nucleation, balances the crystallization rates of I/Br components, and reduces vertical halide phase segregation and residual stress. Experiments show that, upon PEAAc incorporation, FAMACsPb(I_1-x_Br_x_)_3_ PSCs with bandgaps of 1.73, 1.80, 1.85, and 1.92 eV achieve PCEs of 21.3%, 19.5%, 18.1%, and 16.2%, respectively, with *V*_OC_ losses reduced to 0.13–0.21 V. Based on this strategy, PO-TSCs achieved a PCE of 24.12% and a *V*_OC_ of 2.197 V. Su et al. modulated PSC crystallization kinetics by introducing triethanolamine borate (TB), effectively mitigating film defects and voltage losses in inverted-structure devices caused by rapid crystallization (Figure 5) [52]. TB forms coordination and hydrogen bonds with Pb^2+^, FA^+^, and I^−^ in the perovskite precursor, inhibiting halide vacancy formation and reducing ion migration, thereby lowering non-radiative recombination. After TB treatment, perovskite films exhibited enlarged grain sizes (average 313 nm), a 3.27-fold increase in XRD peak intensity, and a reduced defect density of 6.75 × 10^15^ cm^−3^. Single-junction PSCs with a 1.65 eV bandgap achieved a PCE of 21.55%, a *V*_OC_ of 1.24 V, and a minimal *V*_OC_ loss of 0.41 V, representing the lowest among analogous devices. Furthermore, TB treatment significantly enhances device stability: unencapsulated devices retain 90% of their initial efficiency after 2400 h in N_2_ atmosphere and exhibit <4% degradation after 288 h of aging at 65 °C.

(4) Interface passivation enhances performance by reducing defects, optimizing energy-level alignment, improving carrier transport efficiency, and increasing device stability. First, chemical passivation repairs undercoordinated Pb^2+^ or halide vacancies, thereby reducing non-radiative recombination losses. Second, energy-level tuning via interface-layer modification optimizes carrier extraction, lowers energy barriers, and mitigates charge accumulation. Furthermore, the passivation layer suppresses ion migration and enhances resistance to moisture and thermal- and photodegradation, thus improving long-term stability. In 2022, Xie et al. incorporated 10 mol% FA^+^ into MA_1.06_PbI_2_Br(SCN)_0.12_ precursors, improving solubility, suppressing Pb(SCN)_2_ aggregation, and promoting uniform PbI_2_ distribution at grain boundaries, thereby effectively passivating defect states and enhancing film phase stability [53]. The optimized films achieved a 17.4% PCE, with a *V*_OC_ of 1.19 V and an FF of 78.4%. Integrating this wide-bandgap front cell with a narrow-bandgap OPV(PM6:CH1007) extending absorption to 950 nm yielded monolithic PO-TSCs with a PCE of 21.2%, which was among the highest reported at the time. Similarly, in 2023, Wang et al. introduced a synergistic surface-passivation strategy using mixed cations (CA^+^/EA^2+^), significantly enhancing device performance and stability. Figure 6 illustrates the complementary defect passivation mechanisms and bonding changes in CA^+^/EA^2+^, along with comparisons of PL intensity and carrier lifetime improvements [17]. Through the complementary action of CA^+^ and EA^2+^, CA^+^ anchors Pb^2+^ and halide vacancies, while EA^2+^ passivates FA/Cs cation defects. Together, they reduce electron and hole trap densities from 6.95 × 10^15^ and 2.41 × 10^16^ cm^−3^ to 2.77 × 10^15^ and 3.87 × 10^15^ cm^−3^, and lower non-radiative recombination rates from 0.80 to 0.11 ns^−1^. This strategy raised the *V*_OC_ of 1.85 eV wide-bandgap PSCs to a record 1.35 V with an FF of 83.29%. Corresponding PO-TSCs achieved a *V*_OC_ of 2.14 V and a PCE of 24.47%, retaining over 90% of initial efficiency after 500 h of MPP tracking or 60 °C aging. Beyond cation passivation, Lv et al. proposed an efficient surface-passivation strategy using the multifunctional molecule S-ethylisothiourea hydrobromide (SEBr) [54]. SEBr simultaneously passivates Pb-I and FA-I terminated surface defects: its -S-, -NH_3_^+^, and =NH groups form stable Pb-S and Pb-N bonds with undercoordinated Pb^2+^, while Br^−^ fills iodide vacancies via Pb-Br-H bonding, significantly reducing defect density. Moreover, SEBr shifts the interfacial Fermi level between perovskite and the electron transport layer from −4.19 eV to −3.92 eV, facilitating carrier separation and collection. Experiments demonstrate that SEBr-treated PSCs with bandgaps of 1.67 eV and 1.77 eV achieve PCEs of 22.47% and 19.90%, *V*_OC_ values of 1.28 V and 1.33 V, respectively, along with pronounced stability improvements. In recent work, Jiang et al. passivated wide-bandgap perovskite surfaces with cyclohexane 1,4-diammonium diiodide (CyDAI_2_), markedly reducing interfacial recombination and boosting *V*_OC_ [55]. Experiments show that CyDAI_2_ treatment raises the *V*_OC_ of wide-bandgap PSCs from 1.25 V to 1.36 V and the PCE from 14.8% to 18.4%. Furthermore, the match between quasi-Fermi level splitting (QFLSPL) and *V*_OC_ is significantly improved, eliminating the QFLSPL-*V*_OC_ mismatch seen in conventional passivation methods. Compared to the trans-CyDAI_2_, cis-CyDAI_2_-treated films exhibit lower trap-state densities (VTFL reduced from 0.24 V to 0.13 V) and decreased mobile-ion densities (from 8.99 × 10^16^ to 7.13 × 10^16^ cm^−3^), thereby significantly suppressing interfacial recombination and ion migration. PO-TSCs passivated with cis-CyDAI_2_ reached a PCE of 26.4% (certified 25.7%) and retained 93% of their initial efficiency after 700 h of continuous illumination in a nitrogen atmosphere.

### 2.2. Optimization in Narrow-Bandgap OPV Sub-Cells

In OPV sub-cells, the active layer serves as the critical region for photoelectric conversion and directly determines the device’s optoelectronic performance and stability. As the site of photogenerated carrier generation, the active layer must be synergistically optimized for optical absorption, charge transport efficiency, and energy-loss control. Moreover, optimizing the interface layers is another key strategy for enhancing device performance. Tailoring the materials and architecture of these layers can improve interfacial contact between the electrodes and active layer, reduce contact resistance, and thus enhance the transport efficiency of photogenerated carriers.

**Figure 7:**
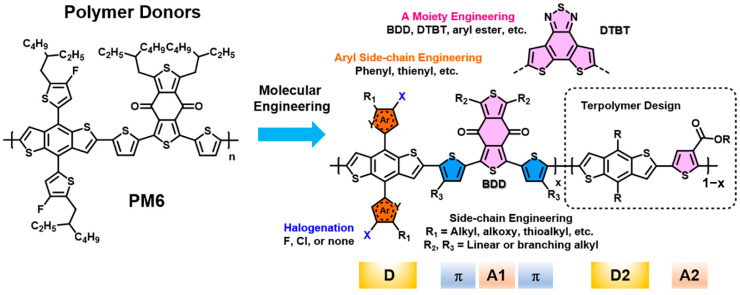
Chemical structure of the polymer donor PM6 and associated molecular-engineering strategies for designing donor–acceptor alternating conjugated copolymers as donor materials paired with NFAs. Reprinted with permission from [64], Copyright 2022, American Chemical Society.

(1) Selection of donor/acceptor materials: The choice and functionalization of the donor are critical: high-molecular-weight polymeric donors readily form interconnecting networks that suppress molecular diffusion and preserve morphological stability, thereby delivering superior device stability compared to small-molecule donors [56,57,58]. The current mainstream donor–acceptor (D-A) alternating conjugated polymers—such as PBDB-T, PM6 (Figure 7), and PM7 [59,60,61]—comprise benzodithiophene (BDT) donor units and benzodithiophene-dione (BDD) acceptor units and have greatly accelerated the rapid development of Y-series non-fullerene acceptors (NFAs). Xin et al. developed the small-molecule donor DR3TBDTC [62] by introducing bulky functional groups to hinder π-π stacking between molecular backbones; devices achieved a PCE of 18.75% and retained 89% of their initial efficiency after 7 days of thermal annealing at 180 °C. Chen et al. designed a D-A block copolymer, PM6-b-PYIT, and applied it in PM6:L8-BO-based OPV [63]. The structural similarity between PM6-b-PYIT and the primary active materials further modulates molecular packing, enhances donor–acceptor compatibility, and improves molecular stacking and charge transport, while also increasing dielectric constant and built-in voltage and suppressing excessive charge recombination.

Prior to the widespread adoption of non-fullerene small-molecule acceptors, fullerene derivatives (e.g., PCBM) dominated the acceptor landscape. These fullerenes exhibit relatively weak optical absorption and limited tunability of energy levels, meaning that the performance of polymer–fullerene blend devices was chiefly constrained by the properties of the polymer donor. In recent years, NFAs have emerged to alleviate these limitations. NFAs combine strong absorption in the visible spectrum, low processing costs, and good solubility with tunable molecular energy levels and bandgaps—enabling higher open-circuit voltages—while the HOMO/LUMO offsets between NFAs and donor materials typically do not compromise charge-separation efficiency. In NFA-based OPV devices, narrow-bandgap acceptors reduce the driving force required for charge generation, thereby effectively enhancing *V*_OC_, and they yield higher photocurrents than fullerene-based systems, resulting in substantial overall performance improvements [65,66,67,68,69].

**Figure 8:**
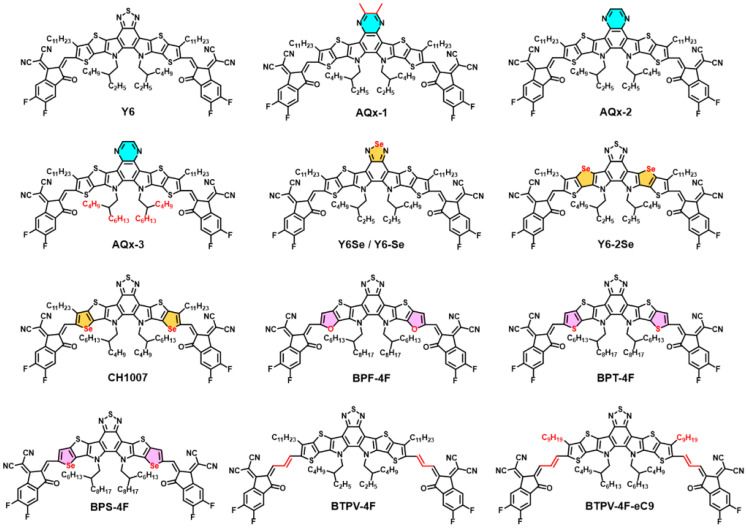
Chemical structures of Y-series acceptor molecules. Reprinted with permission from [64], Copyright 2022, American Chemical Society.

The A-DA′D-A architecture of NFAs adopts a crescent-shaped molecular geometry that promotes tight intermolecular packing into three-dimensional networks, thereby facilitating efficient charge transport and boosting the PCE of OPV sub-cells [70,71]. In cutting-edge research on A-DA′D-A structured NFAs, the Y-series exhibits distinctive advantages: high luminescence efficiency, strong and broad optical absorption, and excellent charge transport properties [72,73,74]. Since Zou et al. first reported the A-D′A-D-A structured Y6 series in 2019, single-junction OPV based on the PM6:Y6 system have rapidly progressed from a PCE of 15.7% to 20% [1,75]. The key unit of Y6 was initially introduced by Cheng et al. in 2011, and subsequent studies have designed additional NFA molecules following the A-DA′D-A motif [76,77,78,79]. The π-conjugated D-A-D core electronic properties in Y-series NFAs can be tailored by varying the electron-donating strength of D units and the electron-withdrawing strength of A′ units, spawning new NFA families such as the Qx and BTz series; Figure 8 illustrates the chemical structures of several representative NFAs. Qin et al. designed and synthesized the novel narrow-bandgap acceptor BTPV-4Cl-eC9 (Eg = 1.22 eV), optimizing its absorption spectrum by incorporating an ethenyl π-bridge and chlorine substituents [37]. Compared to its fluorinated analogue BTPV-4F-eC9 (absorption peak at 891 nm), BTPV-4Cl-eC9 exhibits a red-shifted peak at 911 nm and extends absorption into the near-infrared region (>1050 nm), significantly broadening the spectral response. When combined with the polymer donor PTB7-Th in a layer-by-layer (LBL) architecture, OPV achieved *J*_SC_ = 28.6 mA cm^−2^, FF = 69.2%, and PCE = 12.6%. Integration with a wide-bandgap perovskite front cell yielded PO-TSCs with PCE = 22.0% and *V*_OC_ = 1.88 V, validating the acceptor’s potential for high-efficiency tandem cells. Liu et al. designed and synthesized two novel acceptors, LBz-F and LBz-Cl, by replacing the L8-BO core with BTz and introducing terminal fluorine or chlorine substituents, resulting in shallower HOMO/LUMO levels and a narrowed optical bandgap from 1.39 eV to 1.33 eV [80]. This design not only extends the absorption range but also reduces energy losses. In PM6:L8-BO binary devices, the inclusion of LBz-F or LBz-Cl increased PCEs from 18.07% to 18.67% and 18.37%, respectively, while maintaining a *V*_OC_ of 0.906 V. Furthermore, when this system was applied in PO-TSCs, small-area devices achieved PCE = 22.11%, *V*_OC_ = 2.076 V, *J*_SC_ = 13.45 mA cm^−2^, and FF = 79.2%. For 1 cm^2^ large-area devices, PCE = 20.18% and *V*_OC_ = 2.057 V.

(2) Introduction of additives: Solvent-additive strategies have been widely adopted over the past decade. By adding high-boiling solvents—such as 1,8-diiodooctane (DIO), chloronaphthalene (CN), or diphenyl ether—into the active layer processing [81,82], researchers can tune the evaporation kinetics of the blend: these additives selectively dissolve certain components and retard primary-solvent evaporation, thereby inducing nanostructures with optimal phase separation. However, this conventional solvent-additive approach suffers from significant limitations. Because DIO and similar additives have boiling points above 200 °C, they cannot be fully removed by standard annealing after spin coating; even at concentrations as low as 0.5 vol%, residual solvent remains trapped within the film. These residues act as sources of energetic disorder, broadening the local density of states and increasing the probability of trap-assisted recombination during charge transport. Experimental characterization indicates that residual DIO can increase non-radiative recombination losses by approximately 60 meV, directly reducing the device *V*_OC_. Worse still, the evaporation kinetics of these additives during film formation are highly sensitive to ambient humidity and temperature fluctuations, resulting in poor batch-to-batch morphological reproducibility, which is a fundamental barrier to large-scale industrial production [83,84,85,86,87]. To address this bottleneck, recent breakthroughs in solid-additive strategies have offered a new paradigm. These novel additives typically comprise functional materials with tailored molecular architectures, such as organic small molecules with high dipole moments or polymers capable of forming hydrogen bond networks. They participate directly in active layer self-assembly via intermolecular non-covalent interactions (e.g., van der Waals forces, π-π stacking, or dipole–dipole interactions), enabling precise control over phase-separation behavior without reliance on solvent-evaporation kinetics [88,89,90]. Compared to traditional solvent additives, solid-additive systems offer multiple advantages: (1) their thermal stability allows complete removal during high-temperature annealing, avoiding energetic disorder from chemical residues; (2) molecular design can impart dual functionality, e.g., cyanated molecules that both tune phase morphology and act as electron-trap passivators; and (3) they feature a wide use window (typically 1–3 wt%) and minimal sensitivity to environmental factors, greatly enhancing process reproducibility for large-scale device fabrication.

**Figure 9:**
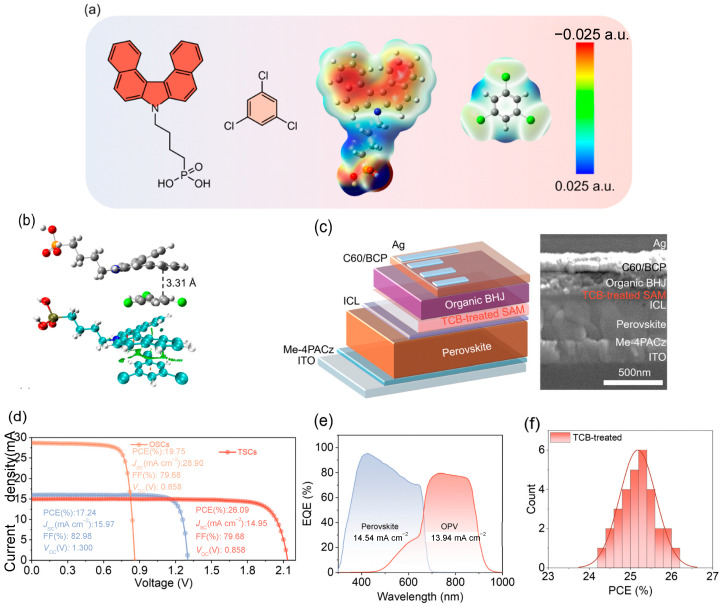
(**a**) Chemical structures of 4PADCB and TCB with their ES distributions; (**b**) visualization of non-covalent interaction analysis; (**c**) schematic of the TSC architecture and corresponding cross-sectional SEM images; (**d**) *J-V* curves of champion OPV, PSCs and TSCs; (**e**) EQE curves of perovskite and organic sub-cells in TSCs; and (**f**) efficiency distribution of 30 individual tandem devices. Reprinted with permission from [92], Copyright 2025, Royal Society of Chemistry.

In 2018, Yu et al. first introduced a volatile solid additive (SA-1) into OPV to optimize device performance [88]. By designing SA-1 with terminal groups structurally analogous to those of the A-D-A acceptor IT-4F, the additive volatilizes during 140 °C annealing, markedly enhancing π-π stacking between acceptor molecules and thus improving charge carrier mobility in the active layer. Experimental results show that devices incorporating SA-1 increased PCE from 12.2% to 13.8% and enhanced electron mobility by an order of magnitude. Compared to conventional solvent additives, SA-1 exhibits superior stability and reproducibility: even with active layer thickness increased to 400 nm, efficiencies remain above 12%. In 2021, Yu et al. introduced a highly volatile, quadrupolar small-molecule additive, DTBF, to achieve precise control over OPV active layer morphology [89]. Experimental data indicate that DTBF significantly enhances molecular ordering and packing density in the active layer, yielding devices with a PCE of 17.1%—compared to 14.8% for untreated cells and 15.6% for those treated with the analogous additive DTB—with a *V*_OC_ of 0.846 V, *J*_SC_ of 26.2 mA cm^−2^, and FF of 77.0%. In 2023, Chen et al. incorporated 1,3,5-trimethoxybenzene (TMB) as a solid additive, significantly optimizing film morphology and suppressing energetic disorder in OPV [91]. TMB acts as a “molecular bridge” by forming strong electrostatic interactions with NFA terminal groups during film formation, inducing precisely controlled π-π stacking distances between adjacent NFA molecules. Devices based on PBDB-TF:eC9 treated with TMB achieved a PCE of 18.61%, with *V*_OC_ increasing from 0.837 V (DIO-processed) to 0.854 V, *J*_SC_ from 26.44 to 27.26 mA cm^−2^, and FF from 79.38% to 79.95%. In 2025, Sun et al. modulated the stacking orientation of the self-assembling molecule 4PADCB by introducing the volatile solid additive 1,3,5-trichlorobenzene (TCB), markedly optimizing hole transport layer performance and thereby enhancing PO-TSC efficiency (Figure 9) [92]. TCB treatment significantly enhances the order of the SAM, increasing the Herman orientation factor from 0.402 to 0.726 while also improving active layer film-formation kinetics and vertical phase separation. Devices based on PM6:BTP-eC9 exhibited a PCE of 20.06% (certified 19.24%) and an FF of 80.64%. When integrated into PO-TSCs, PCE rose to 26.09%, *V*_OC_ to 2.131 V, *J*_SC_ to 14.95 mA cm^−2^, and FF to 81.90%.

(3) Ternary blending systems: In OPV sub-cells, conventional narrow-bandgap organic materials primarily absorb light in specific spectral regions, leading to significant limitations in wide-spectrum energy collection for devices based on single donor–acceptor combinations. Specifically, although narrow-bandgap organic semiconductors effectively absorb photons in the visible region, their photoresponse in the near-infrared and other long-wavelength regions is markedly reduced, and this inherent spectral limitation directly prevents the device from approaching its theoretical solar-harvesting efficiency. To overcome this bottleneck, researchers have recently proposed an innovative ternary-blend design strategy: introducing a third component with complementary optical properties into conventional binary active layers to construct a multidimensional synergistic photovoltaic system. By carefully selecting the third component’s energy-level alignment and spectral characteristics, ternary devices achieve more comprehensive coverage of the solar spectrum. The third component not only fills the optical absorption gaps of the donor–acceptor materials but also, through its unique molecular structure, creates cascade energy-level alignments with the host system, significantly reducing exciton recombination during transport. More importantly, the inclusion of the third component enables modulation of the active layer’s micro- and nanostructures: by tailoring intermolecular π-π stacking and phase-separation morphology, a more optimized three-dimensional charge transport network can be established. This structural refinement ensures efficient exciton dissociation and directional transport of free charge carriers, while also enhancing the active layer’s resilience to photonic and thermal stress [93,94,95].

**Figure 10:**
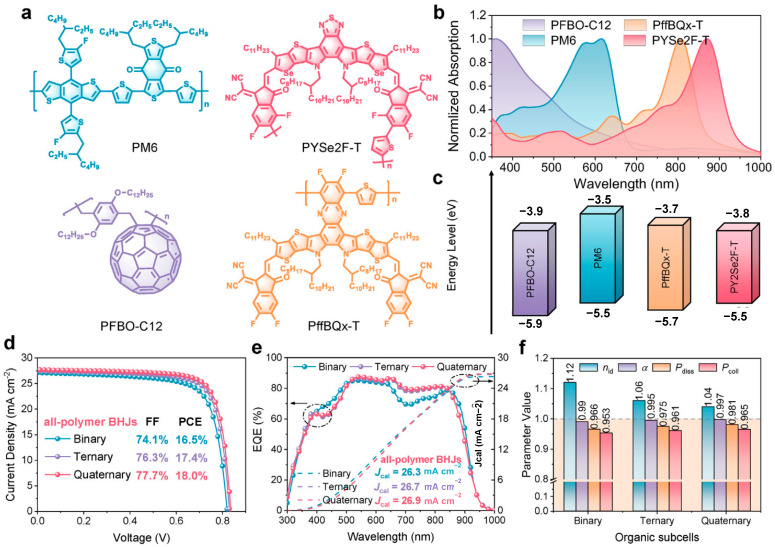
(**a**) Chemical structures of PFBO-C12, PM6, PffBQx-T, and PYSe2F-T; (**b**) normalized UV−Vis absorption spectra; (**c**) energy-level diagram; (**d**) *J-V* curves of binary, ternary, and quaternary all-polymer solar cells; (**e**) EQE response; and (**f**) summarized device parameters. Reprinted with permission from [97], Copyright 2025, Wiley.

In 2024, Cui et al. fabricated ternary organic solar cells using the polymer donor D18 in combination with the non-fullerene acceptors L8-BO and BTP-eC9-4F [96]. By leveraging complementary absorption to extend near-infrared harvesting, these devices increased *J*_SC_ from 25.5 mA cm^−2^ in binary cells to 26.9 mA cm^−2^ and achieved a PCE of 19.2%. When these optimized ternary OPV sub-cells were employed as the rear cell in conjunction with a wide-bandgap perovskite front cell, the resulting PO-TSCs achieved a PCE of 24.5%, a *V*_OC_ of 2.20 V, a *J*_SC_ of 13.8 mA cm^−2^, and an FF of 80.6%. In 2025, Wang et al. designed a quaternary all-polymer bulk heterojunction by incorporating PffBQx-T as a guest polymer and PFBO-C12 as a polymer–fullerene additive into PM6:PYSe2F-T, markedly enhancing the efficiency and stability of PO-TSCs (Figure 10) [97]. By optimizing spectral absorption, suppressing exciton recombination, and facilitating charge transport, the OPV sub-cells achieved a PCE of 18.0%. Devices based on this quaternary all-polymer heterojunction reached a PCE of 24.8%, a *V*_OC_ of 2.13 V, a *J*_SC_ of 14.3 mA cm^−2^, and an FF of 81.7%. Unencapsulated devices stored at approximately 50% relative humidity for 500 h retained 88% of their initial efficiency, while the OPV and PSC sub-cells maintained 77% and 49% of their initial efficiencies, respectively. This exceptional environmental stability is attributed to the hydrophobicity and dense morphology of the all-polymer heterojunction, which effectively suppresses water and oxygen ingress and reduces exciton recombination.

### 2.3. Design of the Interconnection Layer

The ICL in PO-TSCs plays a crucial role: it not only bridges the front and rear sub-cells but also optimizes overall device performance by regulating charge carrier transport and recombination. The ICL typically comprises an electron transport layer (ETL), a hole transport layer (HTL), and an ultra-thin intermediate metal or metal oxide layer [21,98]. An effective ICL should possess the following characteristics: (1) high optical transparency, particularly in the near-infrared region, to suppress parasitic absorption and minimize *J*_SC_ loss in the bottom sub-cell; (2) the ability to form ohmic contacts for efficient charge transport while providing sufficient recombination sites to ensure carriers from each sub-cell recombine effectively within the recombination layer; and (3) resistance to non-orthogonal solvents to prevent solvent-induced damage to the bottom sub-cell. Additionally, the ETL and HTL can serve as barrier and solvent-resistant layers, respectively, preventing moisture and oxygen ingress and protecting the underlying sub-cell from solvent damage after deposition [99,100,101].

Ultra-thin metals such as Ag, Cu, and Au exhibit excellent electrical conductivity and are commonly used as intermediate metal layers; however, their strong self-absorption reduces the optical absorption of the rear sub-cell, resulting in significant optical losses [14,16,102]. In contrast, sputtered transparent conductive oxides (e.g., indium tin oxide (ITO) and aluminum-doped zinc oxide) have emerged as advantageous alternatives. In 2022, Chen et al. developed an ICL based on a 4 nm-thick sputtered IZO layer [21], markedly enhancing both the efficiency and stability of PO-TSCs. They found that the IZO-based ICL exhibits approximately 20% higher transmittance in the near-infrared region compared to a conventional 1 nm Ag-based ICL, thereby reducing current loss in the OPV sub-cell. Experimental data show that devices with the IZO-based ICL achieved a PCE of 23.6% (certified 22.95%), whereas those with the Ag-based ICL reached only 19.5%. Furthermore, the IZO-based ICL demonstrates optimal performance at a thickness of 4 nm, with balanced vertical and lateral conductivities that effectively reduce leakage current and improve FF. In stability tests, IZO-based ICL devices retained 90% of their initial efficiency after 500 h of continuous illumination, while Ag-based ICL devices exhibited more rapid performance degradation. These results indicate that the optical and electrical optimization of IZO-based ICLs provides substantial improvements in both efficiency and stability for PO-TSCs. However, the high-energy particles used in sputtering transparent conductive oxides inevitably damage the underlying layers, leading to device performance degradation. Therefore, it is imperative to employ sputter-protection layers (SPLs) to safeguard the front sub-cell. However, the commonly used BCP exhibits poor thermal stability and is damaged by high-energy sputtered particles during deposition, resulting in performance loss [103,104]. Ma et al. designed and synthesized a thermally crosslinked polymer protection layer, C-C1-P, with excellent thermal stability, significantly enhancing device efficiency and stability [105]. They replaced the conventional BCP layer with the C1 molecule, featuring a styryl group and a phenanthroline backbone. The styryl group undergoes thermal crosslinking to form a dense protective layer that shields the perovskite from high-energy ITO sputtering particles, while the phenanthroline backbone, with its high electron mobility, facilitates interfacial charge transport and reduces non-radiative recombination. Experimental results show that C1 increased the PCE of 1.77 eV PSCs from 16.16% to 18.05% and significantly suppressed thermal degradation, maintaining 91% of the initial efficiency after 200 h at 85 °C. Simultaneously, the use of high-transmittance sputtered ITO (6 nm) as a composite electrode, replacing conventional Ag, reduced optical absorption losses and improved current balance. Combined with the optimized organic heterojunction (PM6: BTP-eC9: PC71BM), the PO-TSCs achieved a PCE of 24.07% (*V*_OC_ = 2.09 V) and retained 92% and 80% of their initial efficiency under high-temperature (85 °C) aging and continuous illumination, respectively. Dong et al. employed polyethyleneimine (PEI) to replace conventional BCP as a protective layer [106]. PEI coordinates with sputtered indium tin oxide nanoparticles to form a conductive composite, significantly enhancing interfacial charge carrier transport while mitigating damage to the PSC sub-cell from high-energy particles. PO-TSCs utilizing PEI/ITO achieved a PCE of 24.33% and retained 98% of their initial efficiency after 2000 h.

**Figure 11:**
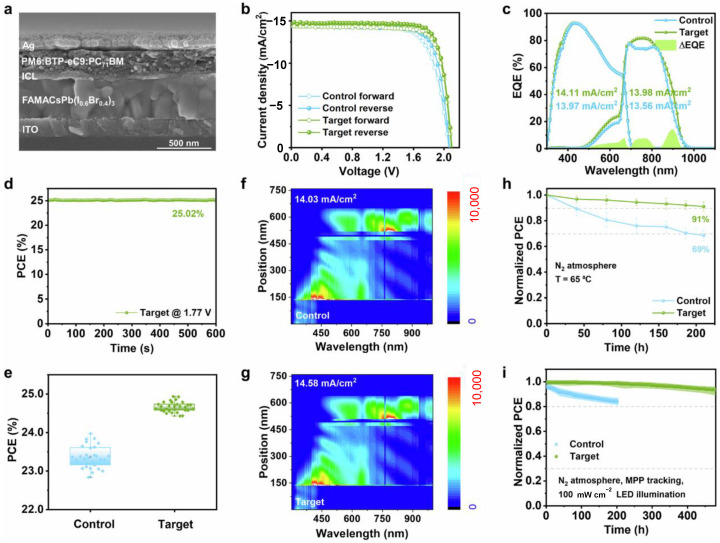
(**a**) Cross-sectional SEM image of the PO-TSCs; (**b**) *J–V* curves of the best PO-TSCs with MoO_3_-based and V_2_O_5_-based ICLs; (**c**) EQE spectra of the perovskite and organic sub-cells for the corresponding PO-TSCs; (**d**) the steady-state power output of the PO-TSCs with V_2_O_5_-based ICL; (**e**) statistics of PCE for 30 PO-TSCs with various ICLs over different batches; (**f**,**g**) distributions of photon absorption in the PO-TSCs with (**f**) MoO_3_-based and (**g**) V_2_O_5_-based ICLs; (**h**) thermal stability of the PO-TSCs with MoO_3_-based and V_2_O_5_-based ICLs stored in N_2_ atmosphere; and (**i**) operational stability of the PO-TSCs with MoO_3_-based and V_2_O_5_-based ICLs under LED illumination. Reprinted with permission from [110], Copyright 2025, Springer Nature.

Beyond sputtered transparent conductive oxides, commonly used ICL materials include p-type metal oxides—NiO_x_, MoO_x_, and VO_x_ (with their doped variants)—and n-type metal oxides—ZnO_x_, TiO_x_, SnO_x_, and WO_x_ (and their doped counterparts). Employing metal oxides as ICL offers enhanced optical transmittance and electrical conductivity. In 2023, Xie et al. proposed a metal-free ICL design based on SnO_x_ for PO-TSCs [107]. The SnO_x_ layer, deposited via atomic layer deposition, not only exhibits high electron transport capability but also acts as a solvent barrier layer protecting the underlying perovskite. Experimental results indicate that the SnO_x_-based ICL achieves substantially higher average transmittance between 700 and 950 nm than conventional metal-based ICLs, thereby reducing current loss in the OPV sub-cell. Furthermore, the SnO_x_ layer markedly suppresses phase separation in wide-bandgap PSCs and passivates interfacial defects, reducing non-radiative recombination losses. By optimizing the SnO_x_ layer thickness to approximately 45 nm, devices achieved a PCE of 22.31%, *V*_OC_ of 2.12 V, *J*_SC_ of 14.08 mA cm^−2^, and FF of 74.95%. In 2024, Wu et al. introduced an optimized ICL design for PO-TSCs [108]. The team incorporated a self-assembled monolayer material (Me-4PACz) to form a bilayer hole-selective interface between MoO_x_ and the organic heterojunction, significantly lowering energetic barriers and enhancing hole extraction. Experimental data show that this design increases *V*_OC_ from 2.10 V to 2.13 V, FF from 79.46% to 82.45%, and *J*_SC_ from 13.73 to 14.00 mA cm^−2^. Moreover, as ICL thickness critically affects carrier transport, the team precisely controlled MoO_x_ thickness to successfully minimize parasitic absorption and electrical losses. At a MoO_x_ thickness of 5 nm, devices exhibited significantly reduced parasitic absorption while maintaining excellent charge transport performance, achieving a peak efficiency with *V*_OC_ at 2.14 V, *J*_SC_ at 14.45 mA cm^−2^, and FF at 81.84%. Ultimately, the optimized tandem devices achieved a PCE of 25.56% (certified 24.65%) and an FF of 83.62%. In 2025, An et al. proposed an ICL design based on a SAM/MoO_3_/SAM trilayer for perovskite/organic tandem solar cells [109]. By introducing self-assembled monolayers on both surfaces of MoO_3_, this design significantly optimizes surface barriers and energy-level alignment, achieving balanced carrier transport and effectively suppressing non-radiative recombination losses. Experimental results demonstrate that this design raises *V*_OC_ from 2.122 V to 2.21 V, *J*_SC_ from 14.11 to 14.42 mA cm^−2^, and FF from 76.46% to 81.75%, culminating in a PCE of 26.05% (certified 24.53%). Moreover, the dark current density decreased from 1.2 × 10^−5^ mA cm^−2^ to 1.8 × 10^−7^ mA cm^−2^, indicating a substantial reduction in non-radiative recombination. The TPV lifetime extended from 5.9 μs to 12.9 μs, further validating the suppression of recombination losses. Device stability also improved significantly, retaining 84% of the initial efficiency after 650 h. Compared to MoO_3_, V_2_O_5_-based ICL offers clearer advantages: in 2025, He et al. proposed a V_2_O_5_-based ICL design for PO-TSCs that significantly reduces optical and electrical energy losses [110]. The team optimized the optical and electrical properties of the V_2_O_5_-based ICL to enable efficient charge collection and current matching, thereby significantly enhancing device efficiency and stability. The V_2_O_5_-based ICL exhibits markedly higher transmittance between 550 and 1100 nm than traditional MoO_3_-based ICL (Figure 11a), particularly in the near-infrared region. This high transmittance enhances the bottom organic sub-cell’s capture of low-energy photons, improving current matching between the sub-cells. Secondly, the V_2_O_5_-based ICL displays higher conductivity (2.23 × 10^−3^ mS cm^−1^) than the MoO_3_-based ICL (1.84 × 10^−3^ mS cm^−1^) (Figure 11b). This enhanced conductivity facilitates rapid, efficient charge transport, reducing recombination and accumulation. Additionally, the hole mobility of the V_2_O_5_-based ICL surpasses that of MoO_3_, further enhancing charge selectivity and extraction efficiency (Figure 11c). In terms of energy-level alignment, the V_2_O_5_-based ICL has a work function of 5.10 eV and a conduction band minimum of 5.52 eV (given its 2.35 eV bandgap). This optimized alignment reduces interfacial Schottky barriers, thereby improving charge collection efficiency (Figure 11d,e). Finally, PO-TSCs employing the V_2_O_5_-based ICL achieved a PCE of 25.1%, *V*_OC_ of 2.10 V, *J*_SC_ of 14.68 mA cm^−2^, and FF of 81.1% (Figure 11f), compared to 23.20% PCE for MoO_3_-based ICL control devices.

In summary, the properties of ICL play a crucial role in the performance and stability of PO-TSCs. The ICL not only requires high optical transparency, particularly in the near-infrared region to minimize parasitic absorption, but also must provide excellent electrical conductivity and appropriate energy level alignment to optimize charge carrier transport and recombination. Studies have shown that optimizing the materials and thickness of the ICL can significantly enhance device efficiency and stability. Furthermore, parasitic light absorption and thickness control are key factors affecting ICL performance and must be precisely optimized to reduce energy loss and improve charge carrier extraction efficiency. Therefore, future research should focus on material selection, thickness optimization, and interfacial energy level alignment of the ICL to further improve the performance and stability of [111,112].

**Table 1:** Summary of 2T PO-TSCs.

Year	Device	*V*_OC_/V	*J*_SC_/(mA cm^−2^)	FF/%	PCE/%	Ref
2022	ITO/NiO_x_/BPA/Cs_0.25_FA_0.75_Pb(I_0.6_Br_0.4_)_3_/C60/ BCP/IZO/MoO_x_/PM6:Y6/Ag	2.06	14.83	77.2	23.60	[21]
2022	ITO/MeO-2PACz/FA_0.8_Cs_0.2_Pb(I_0.5_Br_0.5_)_3_/PC61BM/AZO/ ALD-SnO_x_/InO_x_/MoO_x_/PM6:Y6/C60/BCP/Ag	2.15	14.0	80.0	24.0	[16]
2023	ITO/ZnO/SnO_2_/MAFm(DMSO)/CsPBI_2_Br/ MAFm(1-Butanol)/PDCBT/MoO_3_/Au/ZnO/BCP/ PM6:CH1007/MoO_3_/Ag	2.10	14.23	77.70	23.21	[113]
2023	ITO/MeO-2PACz/FA_0.8_Cs_0.2_Pb(I_0.6_Br_0.4_)_3_/C60/ C-C1-P/ITO/MoO3/PM6:BTP-eC9:PC71BM/TPMA/Ag	2.09	14.58	78.99	24.07	[106]
2023	ITO/NiO_x_/2PACz/perovskite/C60/BCP/Au/MoO_x_/PM6:PM7:Y6:PC71BM/C60/BCP/Ag	2.14	14.17	80.71	24.47	[17]
2024	ITO/NiO_x_/4PADCB/FAMACsPb(I_0.5_Br_0.5_)_3_/PC60BM/C60/SnO_x_/Au/PEDOT:PSS/ D18:L8-BO:BTP-eC9-4F/PDINN/Ag	2.20	13.8	80.6	24.5	[96]
2024	ITO/NiO_x_/2PACz/perovskite/C60/BCP/Au/MoO_x_/ PM6:Y6:PC71BM/C60/BCP/Ag	2.12	14.36	81.65	25.06	[23]
2024	ITO/NiO_X_/Me-4PACz/perovskite/PCBM/AZO/ ITO/MoO_x_/PM6:BTP-eC9/PDINN/Ag	2.144	14.65	80.02	25.13	[114]
2024	ITO/DC-PA/Cs_0.2_FA_0.8_Pb(I_0.6_Br_0.4_)_3_/C60/BCP/Au/MoO_x_/ PM6:Y6:PC71BM/PNDIT-F3N/Ag	2.151	14.36	81.65	25.22	[50]
2024	ITO/CbzNaph/perovskite/C60/BCP/Au/MoO_x_/PM6:BTP-eC9/PDNIT-F3N/Ag	2.15	14.68	81.03	25.54	[18]
2025	m-FTO/M-SQDs/CsPbI_2_Br/PTQ10/MoO_x_/Ag/ZnO/PFN-Br/PM6:BTP-eC9/MoO_x_/Ag	2.21	14.51	80.77	25.90	[115]
2025	ITO/Me-4PACz/perovskite/C60/BCP/Ag/MoO_3_/ TCB-treated SAM/PM6:BTP-eC9/C60/BCP/Ag	2.131	14.95	81.90	26.09	[92]
2025	ITO/NiO_x_/2PACz/perovskite/C60/BCP/Ag /MoO_3_/2PACz/PM6:D18:BTP-eC9/C60/BCP/Ag	2.148	14.60	83.38	26.15	19

## 3. Conclusions and Outlook

In this review, we summarized the recent advances in PO-TSCs, Table 1 shows the research achievements in recent years. The regulatory strategies for wide-bandgap PSC sub-cells and narrow-bandgap OPV sub-cells, as well as the development of the ICL, were discussed. The *V*_OC_ loss in wide-bandgap PSC sub-cells remains a primary bottleneck limiting further performance enhancement of PO-TSCs. This issue can be alleviated through additive engineering and interface passivation strategies, which significantly reduce bulk and grain boundary defects, thereby suppressing non-radiative recombination and minimizing *V*_OC_ loss [116].

As a representative of third-generation photovoltaic technology, PO-TSCs have achieved a breakthrough in PCE, reaching 26.4% through band engineering and material innovation 19. This advancement benefits from the optimized improvements of wide-bandgap PSC sub-cells, the ICL, and narrow-bandgap OPV sub-cells. With ongoing research on perovskite and narrow-bandgap organic donor–acceptor materials, as well as continuous optimization of sub-cells, the ICL, and optical management strategies, PO-TSCs are expected to develop rapidly, surpassing the Shockley–Queisser limit of single-junction solar cells. For ultimate commercialization, narrowing the efficiency gap between laboratory-scale and large-scale production while enhancing long-term operational stability is crucial, particularly from a scalability perspective. PO-TSCs have already demonstrated potential for application in ultra-thin and large-area modules. Therefore, future research should not only focus on improving the efficiency and lifetime of perovskite and OPV sub-cells and developing innovative ICL structures and materials but also explore scalable manufacturing approaches.

Despite significant progress, the performance of PO-TSCs still lags behind that of other comparable perovskite tandem solar cells. Performance-wise, the *V*_OC_ and FF of PO-TSCs are comparable to their counterparts; however, the relatively large bandgap (~1.33 eV) of NFAs in current narrow-bandgap OPV sub-cells results in limited near-infrared absorption, leading to reduced *J*_SC_. Although recent studies have developed ultra-narrow-bandgap NFAs with bandgaps below 1.25 eV, these materials still suffer from low EQE and high energy losses, preventing them from fully replacing the Y-series NFA acceptors. To address this issue, designing novel NFAs with near-infrared spectral response beyond 1000 nm, high EQE, and low energy loss is crucial. Moreover, the problem of photo-oxidative degradation in organic materials remains unresolved, posing a limitation to PO-TSCs. Elucidating the mechanisms of photo-oxidative degradation in organic materials and developing novel organic materials with enhanced photo-oxidative stability will be key future optimization directions for OPV sub-cells. Perovskite components are prone to ion migration and phase segregation under humid and thermal stress, causing significant *V*_OC_ loss and thereby impairing tandem device performance. This necessitates minimizing grain boundaries and passivating surrounding trap states to reduce ion and defect diffusion, thereby suppressing phase segregation in PSC sub-cells through various engineering approaches. The interconnecting layer significantly influences the FF, *J*_SC_, and *V*_OC_ of monolithic tandem devices. The development of novel interconnecting layers should aim for high optical transmittance, ohmic contact with adjacent layers, and protective barrier properties. Excess charge carriers can recombine within the interconnecting layer to ensure balanced charge transport.

## Data Availability

No new data were created or analyzed in this study.
